# Association of ultra-processed food intake with severe non-alcoholic fatty liver disease: a prospective study of 143073 UK Biobank participants

**DOI:** 10.1016/j.jnha.2024.100352

**Published:** 2024-09-27

**Authors:** Yi-Feng Zhang, Wanning Qiao, Jinhong Zhuang, Hanxiao Feng, Zhilan Zhang, Yang Zhang

**Affiliations:** a School of Public Health (Shenzhen), Sun Yat-sen University, Shenzhen, Guangdong, China; b Guangdong Provincial Key Laboratory of Diabetology, Guangzhou Key Laboratory of Mechanistic and Translational Obesity Research, The Third Affiliated Hospital of Sun Yat-sen University, Guangzhou, Guangdong, China

**Keywords:** Ultra-processed food, Non-alcoholic fatty liver disease, Cohort, Dietary intake

## Abstract

**Background:**

Previous studies indicate a link between non-alcoholic fatty liver disease (NAFLD) and unhealthy dietary patterns or nutrient intake. However, it remains unclear whether ultra-processed foods (UPF) contribute to an increased risk of NAFLD. This study aimed to explore how ultra-processed food consumption correlates with severe NAFLD using the UK Biobank data.

**Methods:**

This prospective cohort study included 143,073 participants from the UK Biobank. UPF consumption levels were determined using the NOVA classification and quantified from 24-h dietary recall data. The association between UPF consumption and severe NAFLD (hospitalization or death) was initially examined using Cox proportional hazards models with intake quartiles. Nonlinear associations were investigated using penalized cubic splines fitted in the Cox proportional hazards models. Adjustments were made for general characteristics, sociodemographic factors, body mass index (BMI), and lifestyle.

**Results:**

Throughout the median follow-up period of 10.5 years, 1,445 participants developed severe NAFLD. The adjusted models indicated a significant increase in severe NAFLD risk in higher UPF intake groups compared to the lowest quartile (HR: 1.26 [95% CI: 1.11–1.43]). Subgroup analysis revealed that individuals with a BMI of 25 or higher were at greater risk in the highest quartile of UPF consumption. Sensitivity analyses yielded results consistent with these findings.

**Conclusion:**

Higher consumption of UPF is associated with an increased risk of severe NAFLD. Reducing the intake of UPF can be a potential approach to lower the risk of NAFLD.

## 1 Introduction

Non-alcoholic fatty liver disease (NAFLD) encompasses a spectrum of liver conditions characterized by significant steatosis in over 5% of hepatocytes, occurring in individuals without heavy alcohol consumption [1]. This spectrum ranges from non-alcoholic fatty liver (NAFL) to its more severe counterpart, non-alcoholic steatohepatitis (NASH). NASH can advance to critical stages such as liver fibrosis, cirrhosis, or hepatocellular carcinoma (HCC) [2]. Moreover, NASH is potentially related to other adverse health outcomes like cancer and cardiovascular diseases [3]. Although the exact pathogenesis of NAFLD remains elusive, it is commonly attributed to abnormal lipid accumulation, potentially leading to lipotoxicity, which can also be closely associated with insulin resistance (IR) and metabolic syndrome (MetS) [[4], [5], [6]]. Dietary intake and nutritional status are widely recognized as crucial factors in managing NAFLD. Epidemiological studies also highlight a correlation between nutrient consumption and the onset and progression of NAFLD [[7], [8], [9]]. In the absence of FDA-approved pharmacological treatments, a comprehensive management strategy for NAFLD includes adjusting dietary structure and targeting weight reduction [10,11].

NOVA, a food classification system, categorizes foods based on their processing characteristics into four groups: unprocessed/minimally processed foods, processed cooking ingredients, processed foods, and UPFs [12]. The consumption of UPFs has surged globally, particularly in high-income countries, where they account for over half of the total dietary energy intake. Notably, there's been a significant increase in UPF consumption in lower-income regions, such as a 70.7% rise in Africa [13]. UPF consumption is positively linked with several health issues, including obesity, metabolic syndrome, cardiovascular diseases, diabetes, and cancer, all of which are closely related to NAFLD development [[13], [14], [15], [16], [17], [18], [19]]. This study aims to explore the association between UPF consumption and NAFLD using the data from the UK Biobank to provide insights into the effective health management of NAFLD.

## 2 Methods

### 2.1 Data source and study population

The UK Biobank is a substantial prospective cohort study, enrolling 502,389 individuals aged between 40 and 69 years from across England, Scotland, and Wales. The recruitment phase spanned from December 19, 2006, to October 10, 2010. During this period, comprehensive baseline data encompassing socio-demographic factors, lifestyle, psychosocial characteristics, and health-related information were collected. In addition, trained staff members conducted physical and anthropometric measurements using standardized procedures. Detailed descriptions of these measurements are available in the UK Biobank's online protocol (https://www.ukbiobank.ac.uk).

**Fig. 1:**
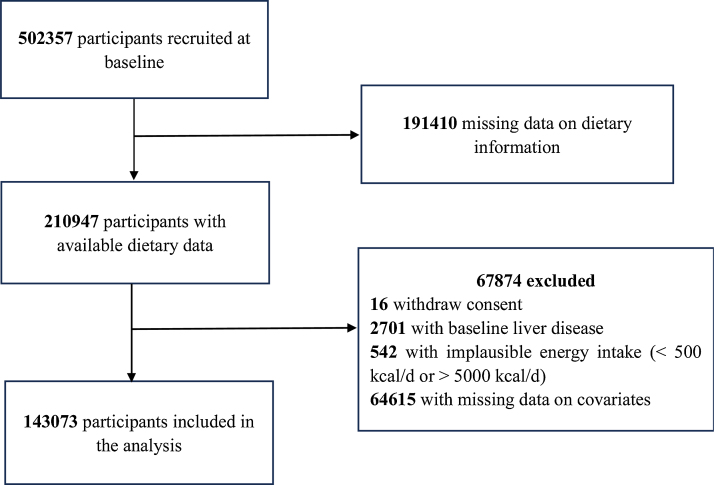
Flowchart for study sample, UK Biobank cohort.

For this research study, we were granted access to the UK Biobank Resource under Application Number 78559 by the UK Biobank’s Access Sub-Committee. We created a cohort for our analysis, excluding participants with missing data on dietary information (*n* = 191410). In addition, exclusions were made for participants who: (1) withdrew consent; (2) had baseline liver disease; (3) reported implausible daily energy intake (<500 kcal/d or >5000 kcal/d); (4) had missing data on essential covariates. This process yielded a final sample size of 143,073 participants for analysis (Fig. 1).

### 2.2 Dietary exposure and degree of food processing

Dietary intakes in our study were assessed using a web-based, self-administered 24-h dietary recall questionnaire (24h-DR). This tool is designed to capture the quantities of over 200 typical food and beverage items consumed in the preceding 24 h. Introduced towards the end of the recruitment phase (2009–2010), this questionnaire was completed online by participants who had provided email addresses. They were invited to fill it out on four separate occasions between 2011 and 2012. This dietary recall method has demonstrated reliability in capturing and estimating food items and nutrient intakes comparable to those obtained through an interviewer-administered 24-h dietary recall [20].

For our study, UPFs were defined following the NOVA classification. The total UPF consumption was quantified based on data collected from the 24h-DR. A detailed list of UPF items is provided in Supplementary Table S1. To calculate the total UPF score, we summed the amounts of all items categorized as UPFs. The primary exposure variable, UPF consumption, was expressed in grams. We computed the average UPF intake using data from at least two 24h-DRs per participant. For food items lacking precise classification, we chose the most frequently consumed option between culinary preparations and manufactured products based on existing data about dietary habits in the UK population. In cases where both alternatives were equally common, we erred on the side of caution by selecting the option with a lower level of processing.

### 2.3 Ascertainment of outcomes

Severe NAFLD was defined based on clinical outcomes, specifically hospitalization or death attributed to NAFLD or NASH. These outcomes were identified through linked hospital and death databases. Participants were systematically followed from their initial visit to the assessment center up to several endpoints: the date of their death, the date of NAFLD or NASH diagnosis, or the last date of available follow-up data (30th September 2021 for England, 28th February 2018 for Wales, and 31 st July 2021 for Scotland), depending on which occurred first.

The criteria for defining severe NAFLD adhered to the International Classification of Diseases, 10th revision (ICD-10). Specifically, cases were identified using ICD-10 codes K76.0 (fatty liver, not elsewhere classified), and K75.8 (NASH, other specified inflammatory liver diseases). This approach aligns with the recommendations of the latest Expert Panel Consensus Statement [21].

### 2.4 Covariates

Baseline study covariates included age, sex, social deprivation (measured by TDI, Townsend Deprivation Index [22]), ethnicity, body mass index (BMI), education, smoking status, frequency of alcohol consumption, and physical activity. Age was calculated from the participant's birth date and the date of their attendance at the assessment center. Ethnicity was categorized into 'white' and 'other'. Educational level was bifurcated into 'higher', encompassing those with college/university degrees or other professional qualifications, and 'lower', for those without such qualifications. Smoking status was self-reported and classified into three categories: never smokers, former smokers, and current smokers. The frequency of alcohol consumption was divided into three groups: never/occasional drinkers, those who drank less than 3 times per week, and those who consumed 3 or more times per week. Physical activity levels were categorized as 'low', 'moderate', or 'high', following the guidelines of the International Physical Activity Questionnaire (IPAQ).

### 2.5 Statistical analysis

Cox proportional hazard models were employed to estimate hazard ratios (HRs) and the corresponding 95% confidence intervals (CIs) for the associations between UPF consumption and severe NAFLD. Follow-up time was utilized as the time metric. The proportional hazards assumption was examined using Schoenfield residual plots, and no violations were found (Supplementary Fig. S1). In continuous models, HRs and 95% CIs were calculated for each absolute 10% increase in the proportion of UPFs in the diet. In the models segmented by UPF consumption quartiles, P values for linear trends were obtained by treating the quartiles as an ordinal variable. The linearity assumption between UPF consumption and the risk of severe NAFLD was verified using restricted cubic spline functions.

Our analysis incorporated five distinct models: Model 1 included only the exposure variable; Model 2 was additionally adjusted for age, sex, and ethnicity; Model 3 further adjusted for the index of multiple deprivation and education level; Model 4 additionally considered body mass index (BMI); and Model 5 was further adjusted for smoking status, frequency of alcohol consumption, and level of physical activity. Subgroup analyses and interaction tests were conducted to identify high-risk subgroups. These were stratified by age (<60 years and ≥60 years), sex (male and female), ethnicity (white and others), smoking status (never, previous, and current smoker), frequency of alcohol consumption (never drink, <3 times/week, and ≥3 times/week), BMI categories (<25, and ≥25 kg/m^2^), and levels of physical activity (low, moderate, and high).

A series of sensitivity analyses were conducted to ensure the robustness of the current study. First, we repeated the analyses excluding NAFLD events occurring in the first two years of follow-up to assess potential reverse causation bias. Second, the analyses were replicated using only the first available 24h-DR to better represent baseline dietary habits.

## 3 Results

### 3.1 Baseline characteristics

A total of 143,073 participants [64,035 (44.8%) men and 79,038 (55.2%) women] were included in the present study. The mean baseline age of study participants was 54.9 (SD: 7.93) years. The average consumption of UPFs was 17.5% of the total diet (SD: 12.2%), with the range spanning from 5.35% (SD: 2.26%) in the lowest quartile to 34.7% (SD: 10.4%) in the highest. Over a median follow-up of 10.5 years (interquartile range: 9.9–11.3 years), 1,945 new cases of severe NAFLD (1.36%) were documented. Table 1 details the baseline characteristics according to UPF consumption quartiles. Notably, higher quartiles correlated with younger age, current smoking status, lower educational levels, higher BMI, and reduced physical activity.

**Table 1:** Characteristics of study participants in the UK Biobank according to quartiles of ultra-processed food intake (N = 143073).

	Quartile 1 (N = 35958)	Quartile 2 (N = 35965)	Quartile 3 (N = 35813)	Quartile 4 (N = 35337)	Overall (N = 143073)	P-value
Proportion of UPF in total diet, %g/day						
Mean (SD)	5.35 (2.26)	11.6 (1.73)	18.7 (2.50)	34.7 (10.4)	17.5 (12.2)	<0.001
Median [Min, Max]	5.67 [0, 8.69]	11.6 [8.69, 14.7]	18.5 [14.7, 23.5]	31.7 [23.5, 100]	14.7 [0, 100]	
Age						
Mean (SD)	56.0 (7.58)	55.7 (7.74)	54.8 (7.97)	53.1 (8.13)	54.9 (7.93)	<0.001
Median [Min, Max]	57.0 [40.0, 70.0]	56.0 [40.0, 70.0]	55.0 [40.0, 70.0]	53.0 [39.0, 70.0]	55.0 [39.0, 70.0]	
Sex						
Male	15807 (44.0%)	16329 (45.4%)	16203 (45.2%)	15696 (44.4%)	64035 (44.8%)	<0.001
Female	20151 (56.0%)	19636 (54.6%)	19610 (54.8%)	19641 (55.6%)	79038 (55.2%)	
Ethnicity						
White	34598 (96.2%)	34815 (96.8%)	34393 (96.0%)	33327 (94.3%)	137133 (95.8%)	<0.001
Others	1360 (3.8%)	1150 (3.2%)	1420 (4.0%)	2010 (5.7%)	5940 (4.2%)	
TDI						
Mean (SD)	−1.60 (2.84)	−1.74 (2.77)	−1.76 (2.77)	−1.58 (2.90)	−1.67 (2.82)	<0.001
Median [Min, Max]	−2.30 [−6.26, 10.3]	−2.44 [−6.26, 10.6]	−2.45 [−6.26, 9.53]	−2.33 [−6.26, 11.0]	−2.38 [−6.26, 11.0]	
Smoking						
Current	2983 (8.3%)	2509 (7.0%)	2511 (7.0%)	2772 (7.8%)	10775 (7.5%)	<0.001
Never	19742 (54.9%)	21056 (58.5%)	21667 (60.5%)	21602 (61.1%)	84067 (58.8%)	
Previous	13233 (36.8%)	12400 (34.5%)	11635 (32.5%)	10963 (31.0%)	48231 (33.7%)	
Education						
Lower	11724 (32.6%)	11563 (32.2%)	12431 (34.7%)	13920 (39.4%)	49638 (34.7%)	<0.001
Higher	24234 (67.4%)	24402 (67.8%)	23382 (65.3%)	21417 (60.6%)	93435 (65.3%)	
Physical						
Low	5688 (15.8%)	6169 (17.2%)	6303 (17.6%)	7149 (20.2%)	25309 (17.7%)	<0.001
Moderate	15116 (42.0%)	15636 (43.5%)	15493 (43.3%)	14652 (41.5%)	60897 (42.6%)	
High	15154 (42.1%)	14160 (39.4%)	14017 (39.1%)	13536 (38.3%)	56867 (39.7%)	
BMI (kg/m^2^)						
Mean (SD)	26.1 (4.11)	26.1 (4.11)	26.4 (4.28)	27.4 (4.93)	26.5 (4.40)	<0.001
Median [Min, Max]	25.5 [14.5, 63.4]	25.5 [14.3, 56.2]	25.8 [12.1, 66.1]	26.7 [12.7, 67.4]	25.9 [12.1, 67.4]	

### 3.2 Association of UPF consumption with severe NAFLD

**Fig. 2:**
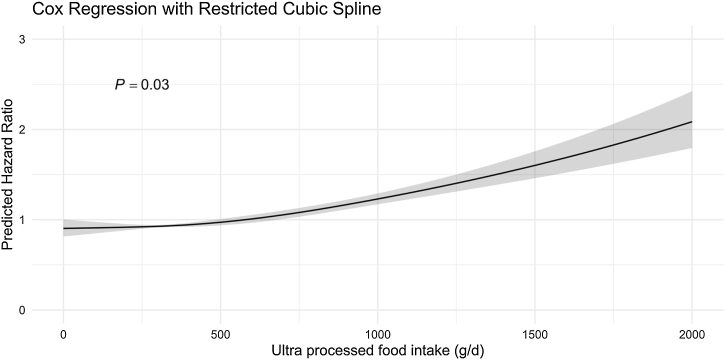
Spline plot for linearity assumption of association between proportion of ultra-processed food in diet and risks of severe NAFLD.

The associations between UPF consumption and severe NAFLD are presented in Table 2. When UPF consumption was treated as a continuous variable, a positive correlation with severe NAFLD risk was observed across all models [HR for an absolute 10% increase in UPF consumption: 1.09 (95% CI: 1.06–1.13); *p* < 0.001]. In the quartile-based analysis, participants in the first quartile (highest UPF consumption) had a 1.38 (95% CI: 1.23–1.60) to 1.49 (95% CI: 1.31–1.69) times higher risk of developing severe NAFLD compared to those in the fourth quartile (lowest consumption), as shown in Models 1 and 2. This association intensified (HR: 1.51; 95% CI: 1.33–1.70) after adjustments for social life factors. The magnitude of the association is slightly attenuated when BMI is included in the further adjustment model (HR: 1.29 [1.14–1.46]). In the fully adjusted model, the association, though reduced, remained statistically significant (HR: 1.26; 95% CI: 1.11–1.43). Notably, while the second and third-quartile groups did not show substantial associations, a statistically significant trend was observed across all four quartiles. Non-linear associations indicated a dose-response relationship between UPF intake and the risk of severe NAFLD, with a notable p-value of 0.03 (Fig. 2).

**Table 2:** Hazard ratio (95% confidence interval) for the associations between ultra-processed food (derived from weight of food consumed) and incidence of severe NAFLD, estimated by multivariable Cox proportional hazards regression.

Number of cases/non-cases	Per 10% absolute increment in ultra-processed food intake	P-value	Quartile of ultra-processed food consumption				P-value for trend
			1st quartile (lowest intake)	2rd quartile	3nd quartile	4st quartile (highest intake)	
Model 1	1.10 (1.10−1.20)	<0.001	1	0.98 (0.86−1.10)	0.98 (0.86−1.10)	1.38 (1.23−1.60)	<0.001
Model 2	1.18 (1.14−1.22)	<0.001	1	0.98 (0.86−1.12)	1.02 (0.89−1.16)	1.52 (1.34−1.71)	<0.001
Model 3	1.17 (1.13−1.21)	<0.001	1	1.00 (0.87−1.14)	1.03 (0.90−1.17)	1.51 (1.33−1.70)	<0.001
Model 4	1.10 (1.07−1.14)	<0.001	1	0.99 (0.87−1.13)	0.99 (0.87−1.13)	1.29 (1.14−1.46)	<0.001
Model 5	1.09 (1.06−1.13)	<0.001	1	0.99 (0.87−1.13)	0.99 (0.87−1.13)	1.26 (1.11−1.43)	<0.001

Model 1 included the exposure variable only.

Model 2 = Model 1 + age (time scale), and stratification by sex and ethnicity.

Model 3 = Model 2 + Index of Multiple Deprivation and education.

Model 4 = Model 3 + BMI continuous at baseline.

Model 5 = Model 4 + smoking status (current/never/previous), alcohol consumption (never/<3 times per week/≥3 times per week), and physical activity level (low/moderate/high).

### 3.3 Subgroup analyses

**Fig. 3:**
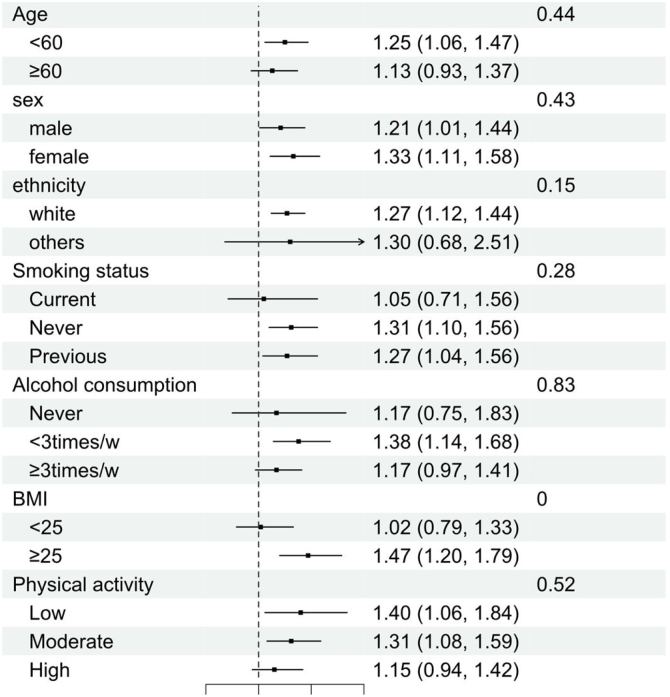
Subgroup analysis of the association between UPFs intake and risk of severe NAFLD.

Fig. 3 illustrates the individual associations for each covariate. There were no significant interaction effects of age, sex, ethnicity, smoking status, alcohol consumption, and physical activity (all *P* for interaction >0.05) on the risk of new-onset severe NAFLD, which to a certain extent indicates that UPFs have a broad range of hazards. Notably, the BMI ≥ 25 group demonstrated a potential impact on the risk of NAFLD onset, corroborating with pathophysiological insights and findings from previous epidemiological studies [23,24]. Moreover, higher alcohol consumption appeared to exhibit a protective effect against severe NAFLD, possibly reflecting the exclusion of participants with liver diseases attributable to alcohol consumption at the baseline.

### 3.4 Sensitivity analyses

In sensitivity analysis, after excluding outcome events that occurred in the first two years of follow-up, the association between UPF consumption and severe NAFLD remained significant, aligning with the results observed in the main analyses (Supplementary Table S4). In contrast, when we limited our analysis to only the first available 24h-DR, the relationship between UPF consumption and severe NAFLD was not statistically significant in the fully adjusted model (Model 5), segmented by UPF consumption quartiles (Supplementary Table S5). However, this association remained significant in the other models.

## 4 Discussion

In this large-scale cohort study leveraging the UK Biobank data, we’ve uncovered a significant link between the consumption of UPFs and the risk of severe NAFLD outcomes for the first time. Our analysis revealed a striking trend: for every 10% increase in UPF consumption, there was a notable rise in severe NAFLD incidence. This trend was particularly pronounced in the fourth quartile, which represented the highest level of UPF intake, compared to lower quartiles. Even after adjusting for several important covariates, this association remained significant. In terms of subgroup analyses, no noteworthy variations (except BMI) were observed, suggesting a uniformly distributed impact of UPFs across different population segments. Our sensitivity analyses, designed to reduce confounding factors, confirmed the consistency of our results across all models, except for Model 5 constructed using only the first available 24h-DR. This consistency across various analytical approaches strengthens the validity of our conclusion that a higher intake of UPFs is associated with an elevated risk of developing severe NAFLD. These insights are particularly relevant in the context of rising UPF consumption globally and its potential public health implications.

The prevalence of NAFLD in Europe is estimated at approximately 23% [25], aligning closely with global figures, and the probability of NASH progressing to cirrhosis ranges between 15 and 20% [26]. Given this widespread prevalence and the limited options for targeted treatments, there is a growing emphasis on research into dietary quality and patterns as crucial strategies for managing the progression of NAFLD. Previous studies, such as those utilizing data from the National Health and Nutrition Examination Survey (NHANES), have indicated a correlation between higher UPF intake and increased NAFLD odds in US adults and adolescents [27,28]. A crucial difference with our research lies in their application of complex logistic regression models in assessing the association. Additionally, NHANES employed methods like Vibration-controlled Transient Elastography (VCTE)-measured Controlled Attenuation Parameter (CAP) or the US Fatty Liver Index for NAFLD assessment, which are arguably more accurate and comprehensive compared to our reliance on admission diagnosis records based on ICD-10 classification. In our study conducted with the UK Biobank data, there may be underreporting of mild NAFLD cases, as only severe NAFLD instances were recorded. This limitation necessitated our focus on severe NAFLD as a representation of the disease's prevalence and impact. Supporting our findings, the China Tianjin Chronic Low-grade Systemic Inflammation and Health (TCLSIH) Cohort Study also presented similar results [29]. A recent systematic review of 15 studies concluded that while larger prospective cohort studies support a positive correlation between UPF intake and the risk of NAFLD and MetS, smaller cross-sectional studies did not consistently demonstrate significant associations. This discrepancy is likely attributable to the methodological limitations inherent in cross-sectional studies. Despite these inconsistencies, the review upheld the view that excessive UPF consumption is a potential risk factor for NAFLD and emphasized the importance of reducing UPF intake among the general adult population to prevent NAFLD and other metabolic diseases [30].

It is important to recognize that the original designation of NAFLD was established without a comprehensive understanding of its underlying pathological mechanisms, leading to a naming convention based on the exclusion of significant contributing factors (i.e., alcohol consumption) [31]. However, recent research has demonstrated that NAFLD is a metabolic disease intricately linked to genetic susceptibility, obesity, nutritional factors, insulin resistance (IR), and metabolic syndrome (MetS) [[32], [33], [34], [35]]. As a result, various position papers and expert consensus statements have advocated for renaming NAFLD as Metabolic-Associated Fatty Liver Disease (MAFLD) [36,37]. More recently, a multi-society Delphi consensus has recommended the term Metabolic Dysfunction-Associated Steatotic Liver Disease (MASLD) to describe this condition, positioning it as a subcategory of Steatotic Liver Disease (SLD). In this framework, alcohol is treated as a conditional variable, and when fatty degeneration is linked to excessive alcohol intake, the condition is termed Metabolic Dysfunction and Alcohol-Associated Liver Disease (MetALD) [38,39]. Notably, 98% of the current NAFLD registry cohort meets the new MASLD criteria [40]. This updated nomenclature more accurately emphasizes the relationship between hepatic fat accumulation and metabolic dysfunction. Consequently, it broadens our understanding of the associations between UPFs and metabolic-related diseases. The meta-analysis indicated that the higher total intake of UPFs is associated with an increased risk of diabetes, hypertension, dyslipidemia, and obesity. However, the quality of evidence, as assessed by the NutriGrade score, is low, and establishing a dose-response relationship between UPF intake and risk levels remains challenging [41]. Similarly, increased intake of processed red meat compared to unprocessed red meat may be potentially positively correlated with the risk of NAFLD [42]. These studies emphasize the need for future research to standardize UPF intake measurement to better understand these associations.

Current research has extensively explored the relationship between various dietary patterns and NAFLD. Several studies across Eurasia have recognized the Mediterranean diet (MED) as a beneficial nutritional approach to managing NAFLD [[43], [44], [45]]. An intriguing cross-sectional study examining religious dietary restrictions within a population following the recognized healthy Mediterranean diet found that both fasters and non-fasters with MetS consumed more UPFs. This finding suggests that excessive UPF consumption may have a broader impact on health, even among fasting populations [46]. Both observational studies [47,48] and clinical randomized controlled trials have consistently indicated a negative correlation between MED adherence and NAFLD, even though many subjects in these studies were overweight or had mild forms of NAFLD [49,50]. Additionally, recent studies suggest that the Dietary Approaches to Stop Hypertension (DASH) dietary pattern may play a preventive role against NAFLD [[51], [52], [53]]. Similarly, diets characterized by anti-inflammatory properties, such as those measured by the Dietary Inflammatory Index (DII) or Energy-adjusted DII, have been linked to a lower risk of severe NAFLD, independent of confounders like pre-existing cardiovascular diseases (CVD) and metabolic syndrome [54,55]. Systematic reviews and meta-analyses echo these findings, highlighting the benefits of other dietary patterns, such as Intermittent Fasting (IF) and Time-Restricted Feeding (TRF), in the context of NAFLD [[56], [57], [58]]. These healthful dietary patterns typically emphasize low-fat, low-salt, and reduced free sugar intake, which can facilitate weight loss - a stark contrast to the characteristics of UPFs, which are often high in fat and salt. This difference in dietary composition and its association with NAFLD progression lend further support to our findings regarding the impact of UPF consumption on the risk of developing severe NAFLD.

Generally, UPFs are typically characterized by their poor nutritional content and high energy density, often being rich in sugars, unhealthy fats, and salt while lacking in dietary fiber, vitamins, proteins, and minerals [59]. These foods contribute to health issues through various mechanisms. For instance, their high red and processed meat content can induce cellular oxidative stress and lipid peroxidation. Moreover, nitrates prevalent in UPFs can be converted into nitrosamines, promoting insulin resistance and diabetes, which are known risk factors for NAFLD [[60], [61], [62]]. Inflammation is a common feature in NAFLD patients, and the pro-inflammatory nutrients in UPFs, such as total, saturated, and trans fats, may exacerbate this condition, thereby contributing to NAFLD onset [63,64]. Additionally, a positive correlation has been observed between serum C-reactive protein, an inflammation marker, and UPF intake, suggesting a link between UPF consumption and systemic inflammation, which could be a potential pathway leading to NAFLD [65].

Experimental studies also suggest that excessive UPF consumption adversely affects gut microbiota, favoring microorganisms that promote inflammation, potentially exacerbating NAFLD through the gut-liver axis [66,67]. In contrast, whole grains, often absent in UPFs, benefit gut flora composition, influencing NAFLD progression [68]. The lack of antioxidants, such as carotenoids and polyphenols, in UPFs further contributes to NAFLD pathogenesis by impeding fatty acid β-oxidation and promoting de novo lipogenesis (DNL) in the liver [69]. The reduced phytochemical content post-grain refining may also impact fat transport through very low-density lipoprotein (VLDL) triglycerides [70]. Beyond nutritional factors, the harmful additives and artificial packaging commonly found in UPFs also significantly affect metabolic health [[71], [72], [73]].

Our study has limitations. Firstly, it relies on the average of 24h-DR, which, while capturing detailed dietary data, may only partially represent long-term dietary patterns. Secondly, food items were categorized into probable groups based on British dietary habits, which could introduce misclassification errors due to limited information on food processing. Thirdly, using the ICD-10 classification for NAFLD diagnosis might result in the absence of mild cases, as such patients may not have hospital records. Therefore, our focus on severe NAFLD might exclude such cases. Lastly, the UK Biobank's demographic composition, primarily middle-aged and white participants, may limit the generalizability of our findings.

In conclusion, this study, conducted within the UK Biobank's framework, unveils a significant association between higher UPF consumption and the risk of severe NAFLD in middle-aged adults. These findings emphasize the need to consider the extent of food processing in dietary habits. Instead of aiming for deeper health literacy and stricter adherence to healthy dietary patterns, reducing UPF consumption might be a practical and effective strategy for NAFLD prevention, promoting a shift toward healthier dietary choices.

## Funding

This work was supported by the 10.13039/501100001809National Natural Science Foundation of China (82000462 and 82170883) (Yang Zhang).

## Author contributions

Conceptualization, Yang Zhang; Data curation, Yi-feng Zhang, Wanning Qiao and Jinhong Zhuang; Formal analysis, Yi-feng Zhang, Wanning Qiao and Jinhong Zhuang; Funding acquisition, Yang Zhang; Investigation, Yi-feng Zhang; Methodology, Yi-feng Zhang, Wanning Qiao, Jinhong Zhuang, Hanxiao Feng and Yang Zhang; Project administration, Yang Zhang; Resources, Yi-feng Zhang and Yang Zhang; Software, Yi-feng Zhang, Wanning Qiao and Yang Zhang; Supervision, Yang Zhang; Validation, Yang Zhang; Visualization, Yi-feng Zhang; Writing – original draft, Yi-feng Zhang, Wanning Qiao, Jinhong Zhuang, Zhilan Zhang and Yang Zhang; Writing – review & editing, Yi-feng Zhang, Wanning Qiao, Jinhong Zhuang, Hanxiao Feng and Yang Zhang.

## Conflict of interest

The authors declare that the research was conducted without any commercial or financial relationships that could be construed as a potential conflict of interest.
