# Supplementary material for: Association of ultra-processed food intake with severe non-alcoholic fatty liver disease: a prospective study of 143073 UK Biobank participants
Source: J Nutr Health Aging. 2024 Sep 27;28(10):100352. doi: 10.1016/j.jnha.2024.100352 (PMC12877288; doi:10.1016/j.jnha.2024.100352)
Supplement: Supplementary file 1 [file mmc1.docx]

**Supplemental Table 1. Food items in each category based on NOVA classifications in the UK Biobank**

| **Nova group** | **Subgroups** |
| --- | --- |
| **Unprocessed or minimally processed foods** | Fruit |
|  | Cereals |
|  | Red meat |
|  | Milk and plain yoghurt |
|  | Fish and seafood |
|  | Vegetables |
|  | Pasta |
|  | Poultry |
|  | Fruit juice |
|  | Roots and tubers |
|  | Eggs |
|  | Legumes and nuts |
|  | Others (coffee, tea, fungi, homemade soup, et.al.) |
| **Processed culinary ingredients** | Butter |
|  | Table sugar |
|  | Plant oil |
| **Processed foods** | Beer and Wine |
|  | Cheese |
|  | Processed bread |
|  | Vegetables and other plant foods preserved in brine |
|  | Parma ham and other salted, smoked or canned meat or fish |
|  | Nuts salted |
| **Ultra-processed foods** | Ultra-processed breads |
|  | Pastries, buns, and cakes |
|  | Milk-based drinks |
|  | Biscuits |
|  | Margarine and other spreads |
|  | Industrial chips (French fries) |
|  | Sausage and other reconstituted meat products |
|  | Breakfast cereals |
|  | Confectionary |
|  | Soft and fruit drinks and fruit juices |
|  | Packaged salty snacks |
|  | Industrial pizza |
|  | Industrial desserts |
|  | Packaged pre-prepared meals |
|  | Sauces, dressing and gravies |
|  | Alcoholic drink |
|  | Other UPFs (chocolate/nut spread, spreadable cheese, sweeteners, meat alternative) |
|  | Other beverages and coffee drinks |

**Supplemental Table 2. Criteria for liver diseases at baseline based on ICD-10 codes in the UK Biobank (data field 41270).**

| **ICD-10 Code** | **Description** |
| --- | --- |
| K76.0 | Fatty (change of) liver, not elsewhere classified |
| K75.8 | Other specified inflammatory liver diseases |
| K74.0 | Hepatic fibrosis |
| K74.1 | Hepatic sclerosis |
| K74.2 | Hepatic fibrosis with hepatic sclerosis |
| K74.6 | Other and unspecified cirrhosis of liver |
| K76.6 | Portal hypertension |
| K76.7 | Hepatorenal syndrome |
| I85.0 | Oesophageal varices with bleeding |
| I85.9 | Oesophageal varices without bleeding |
| I86.4 | Gastric varices |
| I98.2 | Oesophageal varices in diseases classified elsewhere |
| I98.3 | Oesophageal varices with bleeding in diseases classified elsewhere |
| R18 | Ascites |
| Z94.4 | Liver transplant |
| C22.0 | Liver cell carcinoma |

**Supplemental Table 3. Criteria for liver diseases at baseline based on self-reported data in the UK Biobank (data field 20002).**

| **Code** | **Description** |
| --- | --- |
| 1136 | Liver/biliary/pancreas problem |
| 1141 | Oesophageal varices |
| 1155 | Hepatitis |
| 1156 | Infective/viral hepatitis |
| 1157 | Non-infective hepatitis |
| 1158 | Liver failure/cirrhosis |
| 1159 | Bile duct disease |
| 1408 | Alcohol dependency |
| 1506 | Primary biliary cirrhosis |
| 1507 | Haemochromatosis |
| 1578 | Hepatitis a |
| 1579 | Hepatitis b |
| 1580 | Hepatitis c |
| 1581 | Hepatitis d |
| 1582 | Hepatitis e |
| 1604 | Alcoholic liver disease / alcoholic cirrhosis |
